# Analysis of mutational and clinicopathologic characteristics of lung adenocarcinoma with clear cell component

**DOI:** 10.18632/oncotarget.8258

**Published:** 2016-03-22

**Authors:** Chang Gu, Xufeng Pan, Rui Wang, Yuan Li, Xuxia Shen, Jianxin Shi, Haiquan Chen

**Affiliations:** ^1^ Department of Thoracic Surgery, Shanghai Chest Hospital, Shanghai Jiao Tong University, Shanghai, China; ^2^ Department of Pathology, Fudan University Shanghai Cancer Center, Shanghai, China; ^3^ Department of Thoracic Surgery, Fudan University Shanghai Cancer Center, Shanghai, China

**Keywords:** lung adenocarcinoma with clear cell component, lung adenocarcinoma, mutations, prognosis

## Abstract

**Introduction:**

Lung adenocarcinoma with clear cell component is extremely rare and the cases reported in literature remain scarce. The biological behaviors, clinicopathologic characteristics, mutational status and prognosis of lung adenocarcinoma with clear cell component are still uncertain.

**Methods:**

Thirty-eight lung adenocarcinomas with clear cell component and 1659 lung adenocarcinomas were subjected to the study. All the corresponding clinicopathologic data, the distributions of relapse-free survival (RFS) and overall survival (OS), and the status of gene mutations were investigated.

**Results:**

Of 1697 adenocarcinomas, 38 (2.2%) had clear cell component. Fifty percent of adenocarcinomas with clear cell component (11/22) harbored *EGFR* mutation, 41 percent (9/22) harbored *KRAS* mutation and 5 percent (1/22) harbored *AKT1* mutation. Univariable analysis revealed that sex, age, tumor stage, tumor size, nodal stage and pathology were all significant predictors of RFS and OS while the tumor size and nodal stage were still significant predictors in multivariable analysis. There were significantly differences in RFS and OS for lung adenocarcinomas with clear cell component compared with those lung adenocarcinomas.

**Conclusions:**

Lung adenocarcinoma with clear cell component is a rare, malignant tumor with poor prognosis and displays more frequent *EGFR* and *KRAS* mutations.

## INTRODUCTION

Lung cancer is the main cancer worldwide in terms of the incidence and mortality [1, 2]. Accounting for nearly 50 percent of all lung cancers, adenocarcinoma remains the most common histologic subtype of lung cancer and the incidence rate continues to rise in virtually all countries [3, 4]. In 2011, a new adenocarcinoma classification was proposed by the International Association for the Study of Lung Cancer (IASLC)/American Thoracic Society (ATS)/European Respiratory Society (ERS), which discontinued the subtype of clear cell adenocarcinoma and recognized it as a feature [3].

Regardless of the amount and size, the term ‘lung adenocarcinoma with clear cell component’ is now regarded as a cytologic feature due to the IASLC/ATS/ERS classification [3]. Adenocarcinoma cells with clear cytoplasm are frequently focal, but they rarely become the predominant component [5]. Many studies have demonstrated that clear cell feature may occur in association with various histologic subtypes even in some esoteric pulmonary tumors such as fetal adenocarcinoma [6–8]. Although clear cell feature is not considered to be a specific histologic pattern, associations with immunohistochemical and molecular characteristics were confirmed [3].

Despite that clear cell feature may occur in multiple histologic subtypes of lung adenocarcinoma, to our knowledge, lung adenocarcinoma with clear cell component is extremely rare and the cases reported in literature remain scarce [9-12]. Furthermore, the biological behaviors, clinicopathologic characteristics, mutational status and prognosis of lung adenocarcinoma with clear cell component are still undetermined. Therefore, we undertook an investigation of lung adenocarcinoma with clear cell component and compared clinicopathologic characteristics between 38 lung adenocarcinomas with clear cell component and 1659 lung adenocarcinomas. We are the first to detect mutational status of lung adenocarcinoma with clear cell component in such a number.

## RESULTS

### Clinicopathologic factors

**Figure 1:**
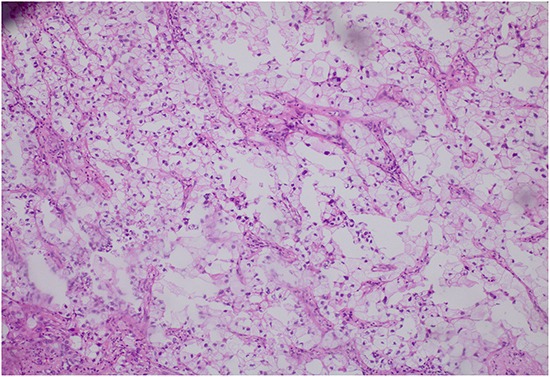
The representative image of lung adenocarcinoma with clear cell component

A total of 1697 patients including 38 lung adenocarcinomas with clear cell component and 1659 lung adenocarcinomas were subjected to the study. Of the 38 lung adenocarcinomas with clear cell component, there were 26 (68.4%) men and 12 (31.6%) women, ranging in age from 30 to 76 years (median, 58.46 years). Apart from five pure clear cell adenocarcinomas confirmed pathologically, the rest 33 specimens of lung adenocarcinoma with clear cell component all contained at least two cell components. The representative image of lung adenocarcinoma with clear cell component is shown in Figure 1 and the characteristics of lung adenocarcinomas with clear cell component are listed in Table 1 and 2. Patients with lung adenocarcinomas consisted of 950 acinar-predominant (57%), 243 solid-predominant (15%), 312 papillary-predominant (19%), 80 lepidic-predominant (5%), 52 mucinous adenocarcinoma-predominant (3%), 18 micropapillary-predominant (1%) and four not detected.

**Table 1:** Clinical characteristics of patients with LAdCC and ADC

Characteristic	LAdCCs(N=38)		ADCs (N=1659)		
	No.	%	No.	%	*P*
Age, yrs					
< 60	21	55.3	383	23.1	**<0.001**
≥ 60	17	44.7	1276	76.9	
Sex					
Male	26	68.4	745	44.9	**<0.001**
Female	12	31.6	914	55.1	
Smoking history					
Never	14	36.8	1141	68.8	0.207
Ever	24	63.2	518	31.2	
Stage					
I-II	23	60.5	1412	85.1	**<0.001**
III-IV	15	39.5	247	14.9	
T-size, cm					
≥3	22	57.9	1284	77.4	**<0.001**
3<, ≥7	13	34.2	353	21.3	
>7	3	7.9	22	1.3	
N Stage					
N0	20	52.6	475	62.2	**<0.001**
N1	7	18.4	85	11.1	
N2	11	28.9	204	26.7	

Abbreviations: LAdCC, lung adenocarcinoma with clear cell component; ADC, adenocarcinoma.

**Table 2:** Clinical details of 22 lung adenocarcinomas with clear cell component

Cases	Age/Sex	Smoking	Stage	Mutations	Subtype
1	52/M	Smoker	IIIa	KRAS(G13C)	A+ Clear cell
2	53/M	Smoker	Ib	KRAS(G12D)+ CTNNB1(T59A)	P+ Clear cell
3	55/M	Smoker	IIIa	KRAS(G13D)	S+ Clear cell
4	74/M	Smoker	IIIa	EGFR(L858R)	S+ Clear cell
5	55/M	Smoker	IIIa	EGFR(L858R)	L+ Clear cell
6	64/M	Smoker	Ia	KRAS(G12C)	A+ Clear cell
7	59/M	Smoker	IIa	KRAS(G13D)	S+ A+ Clear cell
8	64/M	Smoker	Ia	EGFR(770 D=>GY)	L+ Clear cell
9	56/M	Smoker	IIIa	KRAS(G12A)	Clear cell
10	68/F	Never	Ib	EGFR(747LREATS deletion 753P=>S)	A+ Clear cell
11	43/F	Never	IIIb	EGFR(746ELREA deletion)	S+ Clear cell
12	55/M	Smoker	IIa	KRAS(G12C)	S+ Clear cell
13	49/M	Smoker	IIIa	KRAS(G12v)	A+ S+ Clear cell
14	30/M	Never	IIb	AKT1(E17K)	Clear cell
15	56/F	Never	IIIa	EGFR(L858R)	A+ Clear cell
16	57/F	Never	Ia	EGFR(G719C, S768I)	Clear cell
17	70/F	Never	IIIa	EGFR(769-770 ins ASV)	A+ Clear cell
18	66/F	Never	Ia	EGFR(L858R)	P+ Clear cell
19	51/F	Never	Ib	EGFR(L858R)	A+ P+ Clear cell
20	58/F	Never	IIIa	Not detected	S+ A+ Clear cell
21	47/F	Never	IIIa	EGFR(746-750ELREA deletion)	A+S+ Clear cell
22	61/M	/	IIIa	KRAS(G12D)	S+ Clear cell

Abbreviations: M, male; F, female; A, Acinar; P, Papillary; S, solid; L, Lepidic; ins, insertion.

Smoking history in lung adenocarcinomas with clear cell component and lung adenocarcinomas did not differ, but the former tended to have younger age (*p* <0.001), more male patients (*p* <0.001), larger tumor size (*p* <0.001), more advanced disease stage (*p* <0.001) and higher nodal stage (*p* <0.001) (Table 1).

### Mutational status of lung adenocarcinomas with clear cell component

Twenty-one of 38 (55.3%) lung adenocarcinomas with clear cell component were detected harboring mutations in our tested genes. Fifty-two percent (11/21) of them harbored *EGFR* mutation, 43 percent (9/21) harbored *KRAS* mutation and 5 percent (1/21) harbored *AKT1* mutation. Of these,one (case 2, Table 2) harbored both *KRAS* mutation (G12D) and *CTNNB1* mutation (T59A). No *ALK*, *RET* and *ROS1* fusions was uncovered (Table 2).

### Survival analysis

Univariable analysis revealed that sex, age, tumor stage, tumor size, nodal stage and pathology were all significant predictors of RFS and OS (Table 3). The tumor size and nodal stage were still significant predictors of RFS and OS in multivariable analysis, while pathology was not (Table 4).

**Table 3:** Univariable analyses for RFS and OS in 1697 patients with resected lung adenocarcinoma

Variable	RFS			OS		
	HR	95% CI	*p*	HR	95% CI	*p*
Age, yrs	0.499	0.417 to 0.598	**<0.001**	0.631	0.493 to 0.808	**<0.001**
Sex	0.645	0.544 to 0.765	**<0.001**	0.512	0.407 to 0.645	**<0.001**
Smoking history	1.449	1.218 to 1.722	**<0.001**	1.681	1.341 to 2.108	**<0.001**
Stage	5.497	4.576 to 6.603	**<0.001**	4.443	3.500 to 5.641	**<0.001**
T-size, cm	2.625	2.262 to 3.047	**<0.001**	2.661	2.196 to 3.224	**<0.001**
N Stage	2.605	2.366 to 2.869	**<0.001**	2.344	2.070 to 2.654	**<0.001**
Pathology	1.763	1.087 to 2.858	**0.021**	2.311	1.326 to 4.031	**0.003**

Abbreviations: RFS, relapse-free survival; OS, overall survival; HR, hazard ratio.

**Table 4:** Multivariable analyses of RFS and OS in 1697 patients with resected lung adenocarcinoma

Variable	RFS			OS		
	HR	95% CI	*p*	HR	95% CI	*p*
Age, yrs	0.837	0.684 to 1.025	0.085	1.186	0.890 to 1.581	0.244
Sex	0.848	0.668 to 1.077	0.176	0.659	0.479 to 0.907	**0.010**
Smoking history	1.179	0.924 to 1.506	0.186	1.121	0.814 to 1.543	0.484
Stage	1.386	0.865 to 2.221	0.175	1.007	0.521 to 1.947	0.983
T-size, cm	2.044	1.737 to 2.405	**<0.001**	2.023	1.628 to 2.513	**<0.001**
N Stage	1.914	1.493 to 2.453	**<0.001**	2.093	1.476 to 2.968	**<0.001**
Pathology	0.952	0.582 to 1.557	0.844	1.230	0.689 to 2.197	0.483

Abbreviations: RFS, relapse-free survival; OS, overall survival; HR, hazard ratio.

**Figure 2:**
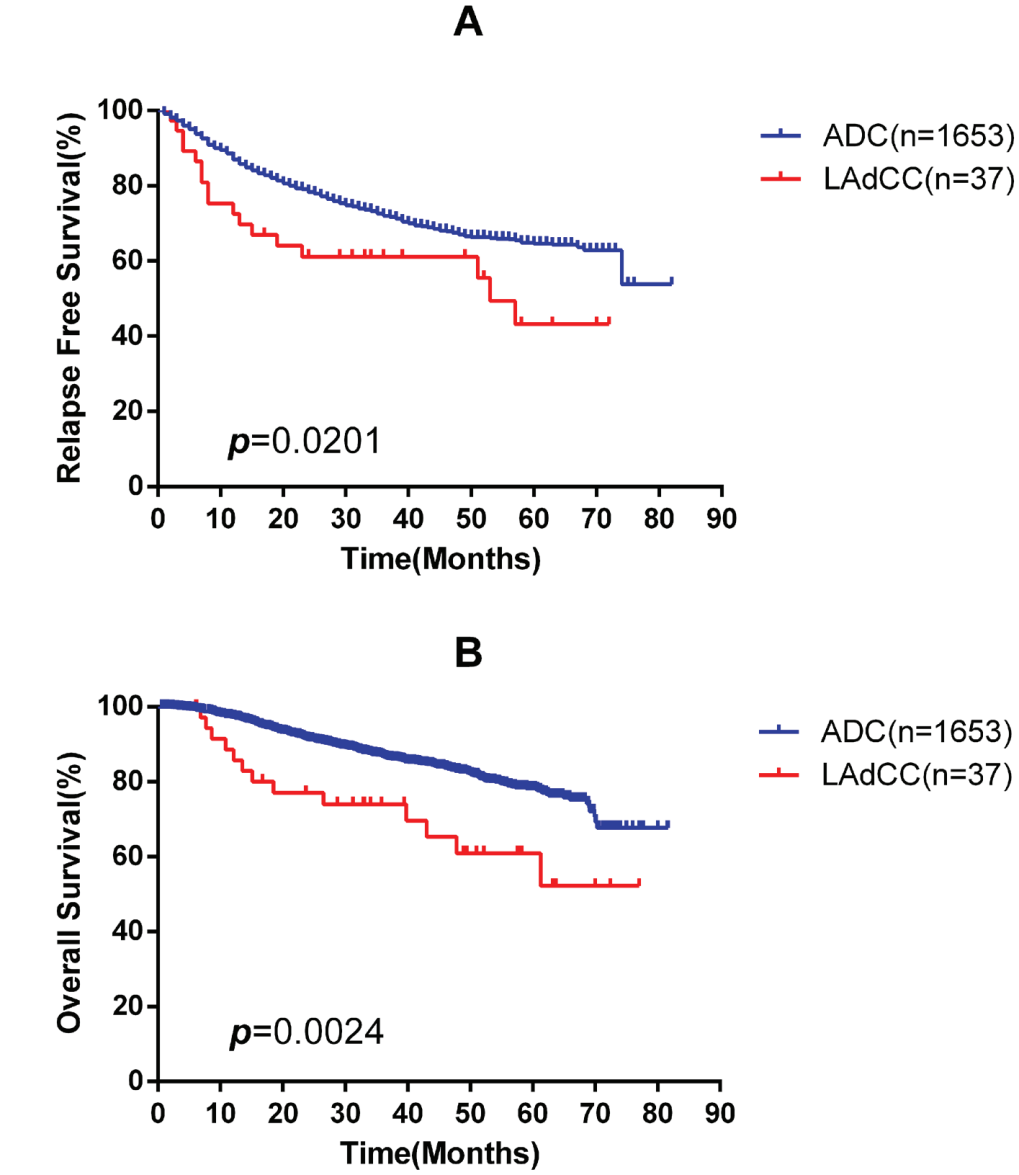
Kaplan-Meier survival curves for relapse-free survival **A.** and overall survival **B.** according to lung adenocarcinoma with clear cell component (LAdCC) and lung adenocarcinoma (ADC).

During the follow-up, 17 (44.7%) lung adenocarcinomas with clear cell component and 525 (31.6%) lung adenocarcinomas experienced a relapse, and finally 13 (34.2%) and 294 (17.7%) patients died, respectively. There were significantly differences in RFS and OS for lung adenocarcinomas with clear cell component compared with those lung adenocarcinomas (Figure 2).

## DISCUSSION

Similar to the 2011 IASLC/ATS/ERS classification, a new classification of lung tumors was proposed by World Health Organization (WHO), which also defined clear cell feature as a type of cytologic characteristics [13]. Though many authors have outlined that clear cell feature can be detected in multiple histologic patterns and may be useful to compare diverse lung tumors, lung adenocarcinoma with clear cell component is extremely rare and the cases reported in literature remain scarce [6–12, 14–16].

In our study, 38 lung adenocarcinomas with clear cell component were identified in1697 patients with primary lung adenocarcinoma (2.2%). The rarity is basically in line with prior studies reported by many authors [7, 17, 18].However, we found a slightly higher incidence rate than what Hinson et al. do (4 of 348, or 1.1%) [16]. Because they excluded the cases with clear cell component less than 50%, whereas we defined lung adenocarcinoma with clear cell component as tumor with clear cell component in at least 5% of the tumor by light microscope according to the new WHO classification.

Lung adenocarcinoma with clear cell component can occur in age groups ranging from 30 to 76 years (median, 58.46 years) with a male predominance (26/38). Lung adenocarcinomas with clear cell component were significantly different from lung adenocarcinomas in RFS and OS (Figure 2). Nevertheless, this histologic pattern only acted as a significant predictor for survival in univariable analysis (Table 3) but not in multivariable analysis (Table 4). The reason may be the fact that those lung adenocarcinoma with clear cell component are associated with advanced disease stage (stage III/IV, 39.5%, Table 1).

Overall, 17 (44.7%) lung adenocarcinomas with clear cell component and 525 (31.6%) lung adenocarcinomas relapsed, and among them, 13 (34.2%) and 294 (17.7%) patients died during follow-up, respectively. Compared with lung adenocarcinomas, the RFS (*p*=0.0201) and OS (*p*=0.0024) of lung adenocarcinomas with clear cell component were significantly inferior (Figure 2). This finding is in agreement with ALA Katzenstein et al, who found 4 lung adenocarcinomas with clear cell component and two of them died within one year [7]. Although a previous study regarded lung adenocarcinoma with clear cell component as a tumor with a relatively good outcome, the study only had one patient and would produce a bias [9].

In consideration of the poor prognosis of lung adenocarcinoma with clear cell component, a thorough treatment strategy should be established. The treatment of clear cell tumor of the lung, a benign tumor, still maintains controversial [19-21]. As for lung adenocarcinoma with clear cell component, the main management for patients with early stage is surgical resection, while multidisciplinary treatment was needed in patients with advanced disease. In our study, we detected mutational status of lung adenocarcinomas with clear cell component. The findings revealed that 21 of 38 (55.3%) lung adenocarcinomas with clear cell component were detected harboring mutations in our tested genes, including 52% (11/21) *EGFR* mutations. And in another study, *EGFR* mutations and *ALK* rearrangements were also found in lung adenocarcinomas with clear cell component [6]. Whether EGFR tyrosine kinase inhibitors or ALK inhibitors are still effective for these special lung adenocarcinomas with clear cell component like lung adenocarcinoma is uncertain and calls for further investigation [13].

Despite clear cell feature is useful to compare diverse lung tumors, the feature can occur in a variety of patterns of lung tumor [14]. It is a challenging work to distinguish lung adenocarcinoma with clear cell component from multiple lung tumors with clear cell component, such as PEComa, benign (with clear cell tumor as a variant), squamous cell carcinoma with clear cell feature, primary clear cell carcinoma and metastases from renal clear cell carcinoma [9, 13, 22]. Differential diagnosis is necessary for these tumors mentioned above and histological and immunochemical tests play important roles. Tumor cells of lung adenocarcinoma with clear cell component may demonstrate finely vacuolated cytoplasm, reveal cytological and histological atypia recognized as lung adenocarcinoma [9, 22]. PEComa exhibits strong cytoplasmic positivity for HMB-45 and is regarded as a benign tumor [23]. Squamous cell carcinoma with clear cell feature can be differentiated on histology and possesses typical immunohistochemical characteristics of squamous cell carcinoma positively expressing squamous markers p40 or p63 [13]. Primary clear cell carcinoma is usually Napsin A and TTF-1 negative [22, 24, 25]. Metastatic renal clear cell carcinoma is CD10 positive and the primary lesion can be found in the kidney by medical imaging technology [26, 27].

There are several limitations in this study. First, despite we detected mutational status of lung adenocarcinomas with clear cell component and reported in such a number, it was still a small sample size. Besides, there were also a few lung adenocarcinomas with clear cell component not included in our study because of the incomplete data. Second, the controversial treatment strategies made us take different options to patients and would lead to diverse outcomes. This could be a potential bias.

In summary, our findings demonstrated that lung adenocarcinoma with clear cell component is a rare, malignant tumor with poor prognosis and displays more frequent *EGFR* and *KRAS* mutations. Multidisciplinary treatment and active postoperative follow-up should be adopted.

## MATERIALS AND METHODS

From June 2007 to March 2012, we consecutively collected resected primary lung tumors as specimens in the Department of Thoracic Surgery of Shanghai Chest Hospital, Shanghai Jiaotong University. We defined lung adenocarcinoma with clear cell component as tumor with clear cell component in at least 5% of the tumor by light microscope and each case was reviewed by our pathologists to confirm the histologic subtypes of resected lung tumors. Besides, all the patients were under routine preoperative testing, including computed tomography (CT) scan, or positron emission tomography-computed tomography (PET-CT), to confirm the diagnosis of primary lung tumor. The combined use of routine preoperative testing and postoperative pathological diagnosis is suggested to make a definite diagnosis of lung adenocarcinoma with clear cell component. Of these, we identified 38 lung adenocarcinomas with clear cell component and 1756 lung adenocarcinomas. A total of 1794 patients were all primary lung adenocarcinoma patients.

Of the 1794 lung adenocarcinomas, 23 patients were excluded for receiving neoadjuvant chemotherapy. 74 patients were excluded because they were lost to follow-up. The remaining 1697 patients including 38 lung adenocarcinomas with clear cell component and 1659 lung adenocarcinomas were subjected to the study.

An informed consent was signed by each patient or legal representative. The study started after obtaining our institutional review board approval. The archives for all patients were reviewed to collect corresponding clinicopathologic data including sex, age, smoking history, pathologic TNM stage (according to the 7th edition of the American Joint Committee on Cancer TNM staging system [28]) and treatment history. The data for disease recurrence and survival were obtained by follow-up clinic or telephone.

### Mutational analysis

The mutational status of *EGFR* (exons 18-21), *KRAS* (exons 2-3), *HER*2 (exons 18-21), *BRAF* (exons 11-15) and *AKT1* (exon 4) was determined using direct dideoxynucleotide sequencing and verified by DNA sequencing analysis. With the use of cDNA, primers was designed to amplify all known fusion variants in order to detect *ALK*, *RET* and *ROS1* fusions. *ALK* fluorescent in situ hybridization (FISH) was used to confirm the accuracy of quantitative real-time reverse transcriptase PCR (qRT-PCR) [29, 30].

### Statistical analysis

All the clinicopathologic data were analyzed using SPSS 19.0 software package (SPSS Inc, Chicago, IL) or Prism 5 (Graph Pad Software Inc., La Jolla, CA). The distributions of relapse-free survival (RFS) and overall survival (OS) were estimated using the Kaplan-Meier method, and the significance between two categories was probed by the log-rank test. The two-tailed significance level was set at *p* <0.05.
